# Hepatitis E Virus and Neurologic Disorders

**DOI:** 10.3201/eid1702.100856

**Published:** 2011-02

**Authors:** Nassim Kamar, Richard P. Bendall, Jean Marie Peron, Pascal Cintas, Laurent Prudhomme, Jean Michel Mansuy, Lionel Rostaing, Frances Keane, Samreen Ijaz, Jacques Izopet, Harry R. Dalton

**Affiliations:** Author affiliations: Centre Hospitalier Universitaire Rangueil, Toulouse, France (N. Kamar, P. Cintas, L. Rostaing);; Université Paul Sabatier, Toulouse (N. Kamar, J.M. Peron, L. Rostaing, J. Izopet);; Royal Cornwall Hospital Trust, Truro, UK (R.P. Bendall, F. Keane, H.R. Dalton);; Centre Hospitalier Universitaire Purpan, Toulouse (J.M. Peron, J.M. Mansuy, J. Izopet);; Centre Hospitalier de Castres, Castres, France (L. Prudhomme);; Health Protection Agency, London, UK (S. Ijaz);; Peninsula College of Medicine and Dentistry, Truro (H.R. Dalton)

**Keywords:** Hepatitis E virus, autochthonous, neurologic symptoms, acute hepatitis, chronic hepatitis, neurologic peripheral symptoms, viruses, synopsis

## MEDSCAPE CME

Medscape, LLC is pleased to provide online continuing medical education (CME) for this journal article, allowing clinicians the opportunity to earn CME credit.

This activity has been planned and implemented in accordance with the Essential Areas and policies of the Accreditation Council for Continuing Medical Education through the joint sponsorship of Medscape, LLC and Emerging Infectious Diseases. Medscape, LLC is accredited by the ACCME to provide continuing medical education for physicians.

Medscape, LLC designates this Journal-based CME for a maximum of 1 *AMA PRA Category 1 Credit(s)*^TM^. Physicians should claim only the credit commensurate with their participation in the activity.

All other clinicians completing this activity will be issued a certificate of participation. To participate in this journal CME activity: (1) review the learning objectives and author disclosures; (2) study the education content; (3) take the post-test and/or complete the evaluation at www.medscapecme.com/journal/eid; (4) view/print certificate.

**Release date: January 25, 2011; Expiration date: January 25, 2012**

## Learning Objectives

Upon completion of this activity, participants will be able to:

- Describe the overall spectrum of neurological manifestations of hepatitis E virus (HEV) infection, based on a case series
- Describe diagnosis of HEV infection in patients who present with neurological symptoms and liver function test abnormalities
- Describe peripheral nerve involvement associated with HEV infection.

## Editor

**P. Lynne Stockton, VMD, MS, ELS(D), **Technical Writer-Editor, *Emerging Infectious Diseases. Disclosure: P. Lynne Stockton, VMD, MS, ELS(D), has disclosed no relevant financial relationships.*

## CME AUTHOR

**Laurie Barclay, MD**, freelance writer and reviewer, Medscape, LLC. *Disclosure: Laurie Barclay, MD, has disclosed no relevant financial relationships.*

## AUTHORS

*Disclosures:* ***Nassim Kamar, MD, PhD; Richard P. Bendall, FRCP; Jean Marie Peron, MD; Pascal Cintas, MD;Laurent Prudhomme, MD; Jean Michel Mansuy, MD; Lionel Rostaing, MD, PhD; Frances Keane, MD, FRCP; Samreen Ijaz, PhD;*** *and* ***Jacques Izopet, PharmD,*** *have disclosed no relevant financial relationships.* ***Harry R. Dalton, FRCP,*** *has disclosed the following relevant financial relationships: served as an advisor or consultant for Beijing Wantai Biological Pharmacy Enterprises Co., Ltd.; GlaxoSmithKline*
