# Supplementary material for: Hepatitis E Virus and Neurologic Disorders
Source: Emerg Infect Dis. 2011 Feb;17(2):173–9. doi: 10.3201/eid1702.100856 (PMC3298379; doi:10.3201/eid1702.100856)
Supplement: Related texts

# Review article: Hepatitis E Virus and Neurologic Disorders

**DOI:** 10.3201/eid1702.100856

**Authors:** Nassim Kamar, Richard P. Bendall, Jean Marie Peron, Pascal Cintas, Laurent Prudhomme, Jean Michel Mansuy, Lionel Rostaing, Frances Keane, Samreen Ijaz, Jacques Izopet, Harry R. Dalton

**Affiliations:** Author affiliations: Centre Hospitalier Universitaire Rangueil, Toulouse, France (N. Kamar, P. Cintas, L. Rostaing);; Université Paul Sabatier, Toulouse (N. Kamar, J.M. Peron, L. Rostaing, J. Izopet);; Royal Cornwall Hospital Trust, Truro, UK (R.P. Bendall, F. Keane, H.R. Dalton);; Centre Hospitalier Universitaire Purpan, Toulouse (J.M. Peron, J.M. Mansuy, J. Izopet);; Centre Hospitalier de Castres, Castres, France (L. Prudhomme);; Health Protection Agency, London, UK (S. Ijaz);; Peninsula College of Medicine and Dentistry, Truro (H.R. Dalton)

## Abstract

Information about the spectrum of disease caused by hepatitis E virus (HEV) genotype 3 is emerging. During 2004–2009, at 2 hospitals in the United Kingdom and France, among 126 patients with locally acquired acute and chronic HEV genotype 3 infection, neurologic complications developed in 7 (5.5%): inflammatory polyradiculopathy (n = 3), Guillain-Barré syndrome (n = 1), bilateral brachial neuritis (n = 1), encephalitis (n = 1), and ataxia/proximal myopathy (n = 1). Three cases occurred in nonimmunocompromised patients with acute HEV infection, and 4 were in immunocompromised patients with chronic HEV infection. HEV RNA was detected in cerebrospinal fluid of all 4 patients with chronic HEV infection but not in that of 2 patients with acute HEV infection. Neurologic outcomes were complete resolution (n = 3), improvement with residual neurologic deficit (n = 3), and no improvement (n = 1). Neurologic disorders are an emerging extrahepatic manifestation of HEV infection.

Hepatitis E virus (HEV) infection is a well-known cause of acute hepatitis in developing countries (*1*). However, autochthonous (locally acquired) HEV infection is also emerging in industrialized countries (*1*), where it is caused by HEV genotype 3 and thought to be a zoonosis transmitted by pigs (*2*). Within the past few years, HEV has been responsible for chronic hepatitis, which can rapidly evolve to cirrhosis, in immunocompromised patients (*3**–**8*). However, little data regarding HEV-related extrahepatic manifestations have been published, although an association between neurologic manifestations (e.g., Guillain-Barré syndrome, neuralgic amyotrophy, acute transverse myelitis) and acute HEV infection has been suggested (*9**–**13*).

Previously, the association between neurologic signs and symptoms and HEV infection has been based on detection of anti-HEV immunoglobulin (Ig) M in serum. However, Rianthavorn et al. reported a case of HEV genotype 3–induced neuralgic amyotrophy in which HEV RNA was detected in the serum of patients with neurologic signs and symptoms (*14*), and we recently detected HEV RNA in the cerebrospinal fluid (CSF) of a kidney-transplant recipient with chronic HEV infection and neurologic signs and symptoms (*15*). We describe 7 cases of HEV-associated neurologic disorders in patients from the Royal Cornwall Hospital, Truro, Cornwall, UK, and Toulouse University Hospital, Toulouse, southwestern France.

In Cornwall, among 55 patients with locally acquired hepatitis E, neurologic signs and symptoms developed among 3 (5.5%). From January 2004 through April 2009, in the organ-transplant unit of Toulouse University Hospital, among 50 solid-organ–transplant patients with HEV, neurologic signs and symptoms developed among 3 (6%). In addition, from January 2005 through December 2009, in the Department of Hepatology of Toulouse University Hospital, among 21 patients with acute HEV infection, neurologic signs and symptoms developed in 1 (4.76%). We describe these 7 cases of HEV-induced neurologic disorders, which occurred in 3 nonimmunocompromised patients with acute HEV infection, in 2 kidney transplant recipients and 1 kidney–pancreas transplant recipient with chronic HEV infection, and in 1 HIV-positive patient with chronic HEV infection (Tables 1, 2).

**Table 1:** Summary of 7 cases of HEV–associated neurologic disorders, Cornwall, UK, and Toulouse, France*

Patient no.	Relevant medical status	HEV infection phase†	HEV genotype	Serum					Cerebrospinal fluid		
				HEV IgG/IgM	HEV RNA	ALT, IU/L†	Bilirubin, µmol/L		HEV RNA	Protein level, g/L	Leukocytes, cells/mm^3^
1	Not immunocompromised	Acute	3e	+/+	+	623	14		–	1.27	145
2	Not immunocompromised	Acute	3e	+/+	+	1160	70		ND	–	–
3	Not immunocompromised	Acute	3f	+/+	+	384	35		–	2	14
4	Kidney–pancreas transplant recipient	Chronic	3f	+/+	+	173	19		+	0.71	1
5	Kidney transplant recipient	Chronic	3f	–/+	+	110	12		+	0.8	8
6	Kidney transplant recipient	Chronic	3f	+/+	+	105	12		+	0.76	7
7‡	HIV positive	Chronic	3a	+/+	+	150	9		+	0.47	1

*HEV, hepatitis E virus; Ig, immunoglobulin; ALT, alanine aminotransferase; +, positive; –, negative; ND, not done. †At time of examination for neurologic symptoms. ‡This patient had positive IgG and IgM Wantai assay results throughout but had negative HEV IgG and IgM results for 3 separate Genelabs assays during 2007–2008.

**Table 2:** Clinical presentation for 7 patients with HEV–associated neurologic disorders, Cornwall, UK, and Toulouse, France*

Patient no.	Neurologic signs and symptoms	Therapy	Outcome
1	Acute inflammatory polyradiculoneuropathy	–	Complete resolution
2	Bilateral brachial neuritis	–	Resolution with residual weakness
3	Guillain-Barré syndrome	IV Ig	Resolution at HEV clearance
4	Ataxia, severe proximal weakness of lower limbs, urine retention, and cognitive dysfunction	IS modification	Resolution with residual motor deficit
5	Encephalitis	IS cessation, foscavir, IV Ig	Complete resolution
6	Peripheral demyelinating polyradiculoneuropathy	IS modification, IV Ig	No improvement
7	Painful sensory peripheral neuropathy	Peg-IFN/ribavirin	Complete resolution

*HEV, hepatitis E virus; –, no specific therapy; IV Ig, intravenous immunoglobulins; IS, immunosuppressant, Peg-IFN, pegylated interferon.

## Methods

The diagnosis of HEV infection was based on the presence of HEV RNA in serum. Serologic analysis showed negative results for hepatitis A, B, and C viruses for all 7 patients and negative HIV results for all but 1 (patient 7). Organ-transplant recipients had negative results for HBV DNA, HCV RNA, and cytomegalovirus (CMV) DNA. Epstein-Barr virus (EBV) DNA was found in the blood of 2 patients (patients 4 and 5).

For the patients from Toulouse, anti-HEV status was determined by using Adaltis EIAgen HEV IgG and IgM kits (Ingen, Chilly Mazarin, France). For patients from the United Kingdom, HEV serology kits from Wantai (Beijing, People’s Republic of China) or Genelabs (Singapore) were used. Serum HEV RNA was detected by real-time PCR with amplification within the open reading frame 2 region (*3**,**5**,**16*). Detected strains were sequenced and compared with reference HEV strains (GenBank) as reported (*5**,**17*).

## The Patients

### Patient 1

A 42-year-old man from Cornwall sought care for severe low-back pain, which progressed to paresthesia in the legs, then the arms, and then weakness with normal sphincter control. The man had not traveled outside the United Kingdom and had had no contact with pigs. Physical examination found weakness of his entire upper limbs and proximal legs. Pinprick sensation was impaired in areas on the right side innervated from C2–4 and distally but asymmetrically in his legs; additionally, S2–5 were involved on the right. Reflexes were diminished or absent in all 4 limbs.

CSF analysis showed high protein levels with lymphocytic pleocytosis (protein 1.27 g/L [reference 0.15–0.45 g/L], glucose 3.5 mmol/L, and leukocytes 145 × 10^9^ cells/L [90% lymphocytes]). Magnetic resonance image (MRI) of the pelvis and lumbar spine showed no abnormalities. Nerve-conduction studies showed distal sensory and motor activity to be within normal limits for all limbs; however, substantial tibial F-wave responses after ankle stimulation were noted, with relative prolongation on the right (right 58.50 milliseconds [ms], left 47.00 ms [reference 52.3 ±4.3 ms, interleg latency difference <5.7 ms]).

Liver function tests showed serum bilirubin within reference range but elevated alanine aminotransferase (ALT) (623 IU/L [reference 3–35 IU/L]). Serologic testing was negative for *Borrelia burgdorferi* and *Treponema pallidum*. Anti-HEV IgM and IgG were detected in the serum, as was HEV RNA, confirming a diagnosis of acute HEV. Molecular characterization showed that the serum HEV was genotype 3e (GenBank accession no. FN869556). CSF was negative for HEV RNA, CMV DNA, EBV DNA, and varicella zoster virus (VZV) DNA.

The best explanation for the clinical and laboratory findings was acute inflammatory polyradiculoneuropathy. The patient was given no specific treatment; neurologic signs and symptoms resolved fully in 3 months, and laboratory parameters returned to reference range within 6 months.

### Patient 2

A 38-year-old man from Cornwall, with type 1 diabetes had a 5-day history of diarrhea, followed by pain, paresthesia, and weakness in his upper arms. He had neither recently traveled outside the United Kingdom nor had contact with pigs. Sensation to pinprick over the C5–6 dermatomes was bilaterally reduced as was strength during elbow flexion, shoulder abduction, and external rotation (worse on the right). Electrophysiologic studies confirmed a diagnosis of bilateral brachial neuritis with denervation of the supraspinatus, infraspinatus, and triceps muscles, which was more severe on the right. CSF was not examined.

Liver function tests showed elevated total serum bilirubin (70 µmol/L [reference 3–17 µmol/L]) and ALT (1,160 IU/L [reference 3–35 IU/L]). Serum was positive for anti-HEV IgG, anti-HEV IgM, and HEV RNA. Molecular characterization showed that the HEV isolated from the serum was genotype 3e (GenBank accession no. FN869555).

The patient was given no specific treatment. Within 6 weeks, liver enzyme levels returned to reference range and HEV RNA became undetectable. Signs and symptoms of brachial neuritis and other neurologic deficits gradually improved over the next 18 months, but residual weakness in his upper right arm remained.

### Patient 3

A 60-year-old woman from Toulouse, France, with type 1 diabetes, had a 1-week history of severe asthenia, jaundice, and progressive weakness in her legs. She had no history of recent travel outside France or contact with animals. She was bedridden with lower limb weakness and complete loss of deep-tendon reflexes but no paresthesia. She had no fever and no biological markers of inflammation, i.e., C-reactive protein was <1 mg/L. CSF protein was 2g/L, glucose 6.2 mmol/L, and leukocyte count 14 × 10^9^ cells/L. Liver function tests showed elevated total serum bilirubin (35 µmol/L [reference 2–21 µmol/L]) and elevated ALT (384 IU/L [reference 5–45 IU/L]). C-reactive protein and creatinine phosphokinase levels were within normal limits. Anti-HEV IgM and IgG were detected in the serum. HEV RNA was also detected in serum and fecal samples, confirming a diagnosis of acute HEV. Molecular characterization showed that the serum HEV was genotype 3f (GenBank accession no. EU 221001.1). CSF was negative for HEV RNA.

The patient’s clinical and laboratory findings are best explained by acute inflammatory demyelinating polyneuropathy (Guillain-Barré syndrome) associated with HEV infection. She was given intravenous immunoglobulin at 0.4 g/kg 1×/d for 5 days. Neurologic signs and symptoms improved rapidly, and liver enzyme levels progressively returned to reference limits within 4 weeks. HEV RNA became undetectable 1 month after initial examination. Her neurologic condition gradually improved over the next 18 months, but residual weakness in her lower limbs remained.

### Patient 4

In a 60-year-old man, acute autochthonous HEV (genotype 3f; GenBank accession no. EU221003) infection developed 27 months after a kidney–pancreas transplant. Acute polyradiculoneuropathy with moderate ataxia and severe proximal weakness of his lower limbs developed 30 months after HEV infection, occurring concomitantly with severe cognitive impairment and intermittent frontal dysfunction. CSF protein was 0.71 g/L, glucose 2.9 mmol/L, and leukocyte count 1 × 10^9^ cells/L. MRI of the cerebrum showed an old lenticular infarction and no acute changes. MRI of the spine showed no abnormalities.

Immunosuppressive therapy for transplantation was a combination of tacrolimus (trough level 6 ng/mL), mycophenolate mofetil, and low-dose prednisolone (5 mg/d). Liver function test results showed total bilirubin within normal limits (19 µmol/L [reference 2–21 µmol/L]) but elevated ALT (173 IU/L [reference 5–45 IU/L]). Liver biopsy sample showed features of chronic active hepatitis; Metavir score was A2F3. CD4 count was 219 × 10^9^ cells/L Serum HEV RNA concentration was 1,572 copies/mL. CSF was negative for anti-HEV IgG but positive for anti-HEV IgM. HEV RNA was detected in CSF obtained at the time of admission. CSF contained no detectable CMV DNA, EBV DNA, Herpes simplex viruses 1 and 2 DNA, VZV DNA, JC virus DNA, cryptococcal antigen, *Toxoplasma gondii* DNA, or *Candida* spp.

Because the patient was aphasic, confused, and drowsy, tacrolimus was replaced by low-dose sirolimus. After 10 days, neurologic signs and symptoms improved. However, 10 months later, despite rehabilitation and physiotherapy, motor deficit in the lower limbs remained and he was still unable to walk. Four months after conversion from tacrolimus to sirolimus, HEV RNA became undetectable in the serum and remains so as of September 2010. The patient declined follow-up lumbar puncture.

### Patient 5

In a 35-year-old man, acute autochthonous HEV (genotype 3f; GenBank accession no. EU220999) infection developed 48 months after kidney transplantation. Three years later, drowsiness and fever (38°C) developed, and neurologic assessment revealed signs and signs and symptoms of encephalitis characterized by confusion and drowsiness without focal signs. CSF protein was 0.8 g/L, glucose 2.5 mmol/L, and leukocyte count 8 × 10^9^cells/L. Initial computed tomographic scan of the brain showed no abnormalities. However, a few hours later, his level consciousness deteriorated and he required mechanical ventilation. Cerebral MRI, performed 24 hours later, showed features of encephalitis with diffuse white matter signal abnormalities in the supratentorial and infratentorial regions.

Immunosuppressive therapy was a combination of tacrolimus (trough level 3 ng/mL), mycophenolate mofetil, and low-dose prednisolone (5 mg/d). Liver function tests showed total bilirubin level within reference range (12 µmol/L [reference 2–21µmol/L]) and an elevated ALT level of 110 IU/L (reference 5–45 IU/L). A liver biopsy sample showed features of chronic active hepatitis; Metavir score was A2F2. CD4 count was 149 × 10^9^ cells/L Serum EBV DNA concentration remained unchanged from 6 months earlier, at 4.24 log_10_ copies/mL. Serum was positive for anti-HEV IgM but negative for anti-HEV IgG. Serum HEV RNA concentration was 2,154,000 copies/mL. CSF was negative for anti-HEV IgG and IgM. HEV RNA and EBV DNA were detected in CSF obtained at the time of admission. CMV DNA, Herpes simplex 1 and 2 DNA, VZV DNA, JC virus DNA, cryptococcal antigen, *Toxoplasma gondii* DNA, and *Candida* spp. were absent in the CSF.

Immunosuppressive therapy was stopped, and Foscavir (6 g/d) and intravenous immunoglobulins (total dose 2 g/kg) were added to the broad spectrum antimicrobial drugs given since admission. MRI showed improvement by day 10, and the patient was extubated. Two months later, despite the absence of neurologic signs and symptoms, CSF protein was 1 g/L, glucose 4.9 mmol/L and leukocyte count 16 × 10^9^ cells/L (96% lymphocytes). HEV RNA and EBV DNA were still detected in the serum and CSF. One year later, HEV spontaneously cleared from serum, but the patient declined a third lumbar puncture.

### Patient 6

In a 44-year-old man from Toulouse, France, acute autochthonous HEV (genotype 3f; GenBank accession no. FJ665423) infection developed 50 months after a kidney transplant (*15*). After 33 months of chronic HEV infection, the patient experienced progressive bilateral muscular weakness, difficulty walking, and palmar and plantar dysesthesia without fever. Neurologic examination revealed peripheral nerve involvement (with proximal muscular weakness that affected all limbs) and central nervous system involvement (bilateral pyramidal signs). Electrophysiologic studies showed signs of peripheral demyelinating polyradiculoneuropathy. MRI of the cerebrum showed no abnormalities. CSF protein was 0.76 g/L, glucose 3.9 mmol/L, and leukocyte count 7 × 10^9^ cells /L.

Immunosuppressive therapy consisted of tacrolimus (trough level 8 ng/mL), mycophenolate mofetil (1 g/d), and low-dose prednisolone (2.5 mg/d). Liver function tests showed total bilirubin level within normal limits (12 µmol/L [reference 2–21]) and elevated ALT level (105 IU/L [reference 5–45 IU/L]). Liver biopsy sample showed cirrhosis; Metavir score was A2F4. Serum CD4-positive count was 167 × 10^9^ cells/L. Serum, but not CSF, contained anti-HEV IgG and IgM. Serum HEV RNA was 260,000 copies/mL, and HEV RNA was detected in CSF. No signs of infection were detected in the serum and CSF, except for EBV DNA, which had remained detectable in the blood since transplantation and at an unchanged concentration of 4.4 log_10_ copies/mL.

After 3 months, because the patient had severe ataxia and loss of sphincter control, neuromuscular biopsy was performed and showed nonspecific signs of neurogenic muscular atrophy but no signs of vasculitis in either muscule or nerve specimens. Consequently, the tacrolimus dosage was markedly reduced to target a trough level of 2.5 ng/mL, and intravenous immunoglobulins were administrated (0.4 g/kg/d for 5 days, total dose 2 g/kg). However, no substantial improvement was observed. After another month, decompensated cirrhosis developed and the patient died of bleeding esophageal varices.

### Patient 7

A 48-year-old man from Cornwall was examined for persistently abnormal liver function that was complicating HIV disease. HIV-1 infection had been diagnosed in 2001 when the patient lived in Cambodia; he was subsequently treated for miliary tuberculosis in 2003.

When back in the United Kingdom, before receiving any antiretroviral medications, the patient had mildly elevated ALT (51 IU/L, reference 3–35 IU/L); other liver enzymes were within reference range. CD4 count was 30 × 10^9^ cells/L, and HIV-1 viral load was 8.3 × 10^4^ copies/mL. Accordingly, in January 2007, the patient was given tenofovir/emtricitabine and lopinavir/ritonavir. In February 2007, the regimen was changed to abacavir/lamivudine and efavirenz; after this time, because the patient had difficulty tolerating these drugs, the regimen was again changed to abacavir/lamivudine and lopinavir/ritoanvirfor, which led to serum HIV RNA clearance in June 2007. In March 2007, ALT had risen to 114 IU/L, but there was no serologic evidence of syphilis or acute hepatitis A, B, or C. From July 2007 through July 2009, ALT remained elevated (118–195 IU/mL). In July 2007, HEV IgM and IgG were detected by enzyme immunoassay. HEV infection was confirmed by detection of HEV RNA (genotype 3a; GenBank accession no. FN869554) in serum. Testing of stored plasma samples for HEV RNA showed that the patient had been viremic since July 2007 and had remained so for 30 months, confirming chronic HEV infection. Liver biopsy sample showed cirrhosis; Metavir score was A3F4. The time HEV infection was acquired and its geographic origin remain uncertain.

In 2005, soon after completing antituberculous chemotherapy, the patient experienced progressive and painful sensory peripheral neuropathy with decreased pinprick sensation and proprioception and weakness in the distal lower limbs. At the time, these neurologic signs and symptoms were thought to have resulted from either HIV-associated neuropathy or previous isoniazid-containing antituberculous chemotherapy. In May 2009, CSF contained 0.47 g protein/L, 3.2mmol glucose/L, 1 × 10^9^ leukocytes/L and HEV RNA.

In July 2009, because of chronic HEV liver infection, the patient was given pegylated interferon-α-2a and ribavirin. During the course of this treatment, the neurologic signs and symptoms improved, and by the time the virus cleared, they were virtually gone. One month after completion of therapy and symptom resolution, CSF levels of protein, glucose, erythrocytes, and leukocytes were within reference range; however, HEV RNA was still detected. An exact estimate of HEV viral load was not performed, but the semiquantitative technique used showed substantial reduction of HEV (barely detectable) in a follow-up CSF sample.

## Discussion

Data about neurologic sequelae of HEV infection are scarce and come mainly from the Indian subcontinent. These data probably refer to HEV genotype 1 infection because this is the predominant genotype in this area.

In industrialized countries, autochthonous HEV infection has been described for a large number of persons who have not traveled to areas where HEV has traditionally been considered endemic (*1*). Hepatitis E for these persons is thought to be a porcine zoonosis and is generally caused by HEV genotype 3 (and genotype 4 in the People’s Republic of China and Japan). The clinical features of hepatitis E in persons in industrialized countries are quite distinct from those in developing countries: HEV occurs most often in middle-aged and elderly men, and associated mortality rate is 5%–10% (*1*). Information about the spectrum and magnitude of disease caused by HEV genotype 3 is still emerging. For example, in recent years chronic HEV infection (with rapid development of cirrhosis) in immunocompromised persons has been demonstrated (*3**–**8*).

For the 7 cases of HEV genotype 3 infection with associated neurologic disorders reported here, the spectrum of neurologic injury associated with HEV infection was quite wide and was found in patients with acute and chronic HEV infection. However, these neurologic signs and symptoms can be divided in 2 clinical pictures. The first and dominant clinical picture is peripheral nerve involvement, which was observed for 5 of the 7 patients. These 5 patients had acute or chronic polyradiculoneuropathy. In these cases, proximal peripheral nerve involvement was similar to that associated with immune or other infectious diseases. In addition to this dominant clinical picture, 1 patient had central and peripheral manifestations, and 1 patient had encephalitis. Only 1 of the 2 patients had fever, and meningitis with lymphocytic CFS was mild or absent in that patient.

For several reasons, we think that the association between HEV genotype 3 infection and the neurologic signs and symptoms in the 7 patients reported here is causal. First, similar neurologic illnesses have been described in 2 clinically and geographically distinct populations. Second, for all patients, the diagnosis of HEV was confirmed by molecular techniques, which excludes the possibility of cross-reacting antibodies causing a spurious association between HEV infection and neurologic illness. Third, HEV RNA was detected in the CSF of some patients. Finally, there was a temporal association between clearance of HEV viremia and resolution of the neurologic signs and symptoms.

The mechanisms of neurologic damage in our patients are unknown. Many viruses (including hepatotropic viruses) trigger neurologic signs and symptoms, especially Guillain-Barré syndrome (*18*). Such infections may elicit an immune response that cross-reacts with axolemmal or Schwann cell antigens and thereby damages peripheral nerves (*18*). Among the 7 cases reported here, HEV RNA was detected in the CSF of 4 patients with chronic HEV infection and neurologic signs and symptoms, suggesting that local viral replication is occurring in the central nervous system, which may cause direct neuronal damage. Additional evidence for viral replication in the central nervous system is the discovery that different HEV quasispecies coexisted in the serum and CSF of a patient with chronic HEV infection (patient 6) (*15*). Neurologic signs and symptoms may result from infection with, or emergence of, neurotropic HEV variants (*15*).

On the basis of our observations, we are unable to estimate how frequently HEV genotype 3 infections cause neurologic damage. In the series of (mainly) acute cases in the United Kingdom, neurologic signs and symptoms were present in ≈5% of patients; in the series of chronic HEV infection in Toulouse, incidence was ≈6%. The true rate of neurologic sequalae associated with HEV 3 infection may be higher because autochthonous genotype 3 infections in industrialized countries are not widely recognized by many clinicians (including neurologists). This may be partly because of the understated clinical presentation of HEV infection. Only 1 patient reported here was icteric at the time of initial examination, and liver function test results of the immunocompromised patients were only modestly elevated. HEV-induced neurologic disorders occurred with 3 subtypes of HEV (i.e., HEV 3a, HEV 3e, and HEV 3f). These data indicate that neurologic injury induced by HEV genotype 3 is not subtype specific; because subtypes a, e, and f are found throughout Europe and North America, the geographic range of disease may well be extensive.

In conclusion, neurologic signs and symptoms are an emerging extrahepatic manifestation of HEV genotype 3 infection. We recommend that clinicians strongly consider the possibility of HEV infection in patients with neurologic disorders, especially those with peripheral nerve involvement and liver abnormalities indicated by blood tests. The diagnosis may be suggested by HEV serology but should be confirmed by molecular documentation of HEV RNA in the serum, CSF, or both.

## References

[1] Dalton HR, Bendall R, Ijaz S, Banks M. Hepatitis E: an emerging infection in developed countries. Lancet Infect Dis. 2008;8:698–709. 10.1016/S1473-3099(08)70255-X18992406

[2] Dalton HR, Stableforth W, Thurairajah P, Hazeldine S, Remnarace R, Usama W, Autochthonous hepatitis E in southwest England: natural history, complications and seasonal variation, and hepatitis E virus IgG seroprevalence in blood donors, the elderly and patients with chronic liver disease. Eur J Gastroenterol Hepatol. 2008;20:784–90. 10.1097/MEG.0b013e3282f5195a18617784

[3] Kamar N, Selves J, Mansuy JM, Ouezzani L, Peron JM, Guitard J, Hepatitis E virus and chronic hepatitis in organ-transplant recipients. N Engl J Med. 2008;358:811–7. 10.1056/NEJMoa070699218287603

[4] Ollier L, Tieulie N, Sanderson F, Heudier P, Giordanengo V, Fuzibet J, Chronic hepatitis after hepatitis E virus infection in a patient with non-Hodgkin lymphoma taking rituximab. Ann Intern Med. 2009;150:430–1.1929308410.7326/0003-4819-150-6-200903170-00026

[5] Dalton HR, Bendall R, Keane F, Tedder R, Ijaz S. Persistent carriage of hepatitis E virus in patients with HIV infection. N Engl J Med. 2009;361:1025–7. 10.1056/NEJMc090377819726781

[6] Gerolami R, Moal V, Colson P. Chronic hepatitis E with cirrhosis in a kidney-transplant recipient. N Engl J Med. 2008;358:859–60. 10.1056/NEJMc070868718287615

[7] Kamar N, Mansuy JM, Cointault O, Selves J, Abravanel F, Danjoux M, Hepatitis E virus–related cirrhosis in kidney- and kidney-pancreas–transplant recipients. Am J Transplant. 2008;8:1744–8. 10.1111/j.1600-6143.2008.02286.x18557740

[8] Haagsma EB, van den Berg AP, Porte RJ, Benne CA, Vennema H, Reimerink JH, Chronic hepatitis E virus infection in liver transplant recipients. Liver Transpl. 2008;14:547–53. 10.1002/lt.2148018383084

[9] Kamani P, Baijal R, Amarapurkar D, Gupte P, Patel N, Kumar P, Guillain-Barré syndrome associated with acute hepatitis E. Indian J Gastroenterol. 2005;24:216.16361768

[10] Sood A, Midha V, Sood N. Guillain-Barré syndrome with acute hepatitis E. Am J Gastroenterol. 2000;95:3667–8.1115192910.1111/j.1572-0241.2000.03409.x

[11] Loly JP, Rikir E, Seivert M, Legros E, Defrance P, Belaiche J, Guillain-Barré syndrome following hepatitis E. World J Gastroenterol. 2009;15:1645–7. 10.3748/wjg.15.164519340910PMC2669950

[12] Fong F, Illahi M. Neuralgic amyotrophy associated with hepatitis E virus. Clin Neurol Neurosurg. 2009;111:193–5. 10.1016/j.clineuro.2008.09.00518986757

[13] Mandal K, Chopra N. Acute transverse myelitis following hepatitis E virus infection. Indian Pediatr. 2006;43:365–6.16651680

[14] Rianthavorn P, Thongmee C, Limpaphayom N, Komolmit P, Theamboonlers A, Poovorawan Y. The entire genome sequence of hepatitis E virus genotype 3 isolated from a patient with neuralgic amyotrophy. [**PMID: 20100114**]. Scand J Infect Dis. 2010;42:395–400. 10.3109/0036554090349655120100114

[15] Kamar N, Izopet J, Cintas P, Garrouste C, Uro-Coste E, Cointault O, Hepatitis E virus–induced neurological symptoms in a kidney-transplant patient with chronic hepatitis. Am J Transplant. 2010;10:1321–4. 10.1111/j.1600-6143.2010.03068.x20346067

[16] Mansuy JM, Peron JM, Abravanel F, Poirson H, Dubois M, Miedouge M, Hepatitis E in the south west of France in individuals who have never visited an endemic area. J Med Virol. 2004;74:419–24. 10.1002/jmv.2020615368508

[17] Legrand-Abravanel F, Mansuy JM, Dubois M, Kamar N, Peron JM, Rostaing L, Hepatitis E virus genotype 3 diversity, France. Emerg Infect Dis. 2009;15:110–4. 10.3201/eid1501.08029619116067PMC2660688

[18] Hughes RA, Cornblath DR. Guillain-Barré syndrome. Lancet. 2005;366:1653–66. 10.1016/S0140-6736(05)67665-916271648

# Review article: Hepatitis E Virus and Neurologic Disorders

**DOI:** 10.3201/eid1702.100856

**Authors:** Nassim Kamar, Richard P. Bendall, Jean Marie Peron, Pascal Cintas, Laurent Prudhomme, Jean Michel Mansuy, Lionel Rostaing, Frances Keane, Samreen Ijaz, Jacques Izopet, Harry R. Dalton

**Affiliations:** Author affiliations: Centre Hospitalier Universitaire Rangueil, Toulouse, France (N. Kamar, P. Cintas, L. Rostaing);; Université Paul Sabatier, Toulouse (N. Kamar, J.M. Peron, L. Rostaing, J. Izopet);; Royal Cornwall Hospital Trust, Truro, UK (R.P. Bendall, F. Keane, H.R. Dalton);; Centre Hospitalier Universitaire Purpan, Toulouse (J.M. Peron, J.M. Mansuy, J. Izopet);; Centre Hospitalier de Castres, Castres, France (L. Prudhomme);; Health Protection Agency, London, UK (S. Ijaz);; Peninsula College of Medicine and Dentistry, Truro (H.R. Dalton)

## Earning CME Credit

To obtain credit, you should first read the journal article. After reading the article, you should be able to answer the following, related, multiple-choice questions. To complete the questions and earn continuing medical education (CME) credit, please go to www.medscapecme.com/journal/eid. Credit cannot be obtained for tests completed on paper, although you may use the worksheet below to keep a record of your answers. You must be a registered user on Medscape.com. If you are not registered on Medscape.com, please click on the New Users: Free Registration link on the left hand side of the website to register. Only one answer is correct for each question. Once you successfully answer all post-test questions you will be able to view and/or print your certificate. For questions regarding the content of this activity, contact the accredited provider, CME@medscape.net. For technical assistance, contact CME@webmd.net. American Medical Association’s Physician’s Recognition Award (AMA PRA) credits are accepted in the US as evidence of participation in CME activities. For further information on this award, please refer to http://www.ama-assn.org/ama/pub/category/2922.html. The AMA has determined that physicians not licensed in the US who participate in this CME activity are eligible for *AMA PRA Category 1 Credits*™. Through agreements that the AMA has made with agencies in some countries, AMA PRA credit is acceptable as evidence of participation in CME activities. If you are not licensed in the US and want to obtain an AMA PRA CME credit, please complete the questions online, print the certificate and present it to your national medical association.

## Article Title: Hepatitis E Virus and Neurologic Disorders

## CME Questions

**1. Based on the above study by Dr. Kamar and colleagues, which of the following statements about the overall spectrum of neurological manifestations of HEV infection is correct?**A. Approximately 15% of patients with HEV infection in this series had neurological manifestationsB. The pattern of neurological involvement was exclusively polyradiculoneuropathy in all casesC. One patient presented with central and peripheral manifestationsD. There was marked cerebrospinal fluid (CSF) lymphocytic meningitis in most cases with central involvement**2. You are asked to consult on a 49-year-old man suspected to have neurological manifestations of HEV infection. He presented with a history of severe low back pain, followed by tingling in the legs and then in the arms, and then followed by limb weakness. Examination showed global arm weakness, proximal leg weakness, patchy loss to pinprick sensation in a radicular pattern, stocking distribution of sensory loss, and globally decreased or absent reflexes. Routine blood chemistries showed normal bilirubin but markedly elevated alanine aminotransferase (ALT). Based on the above study, which of the following statements would be most likely to be correct regarding his diagnostic workup?**A. HEV serology is sufficient to confirm the diagnosisB. Diagnosis should be confirmed using molecular techniques to document HEV RNA in the serum and/or CSFC. MRI of the lumbar spine is likely to be abnormalD. Nerve conduction velocity testing is likely to be completely normal**3. The patient in Question 2 has HEV RNA in the serum and CSF and is diagnosed with acute inflammatory polyradiculoneuropathy secondary to HEV genotype 3 infection. Based on the above study, which of the following statements about his condition is most likely correct?**A. Proximal peripheral nerve involvement does not resemble that seen in most immune disordersB. HEV infection may elicit an immune response that cross-reacts with axolemmal or Schwann cell antigens, thereby damaging peripheral nervesC. The patient's condition is likely to rapidly deteriorate, causing deathD. Steroid treatment is recommended

## Activity Evaluation

**Table:** 

1. The activity supported the learning objectives.				
Strongly Disagree				Strongly Agree
1	2	3	4	5
2. The material was organized clearly for learning to occur.				
Strongly Disagree				Strongly Agree
1	2	3	4	5
3. The content learned from this activity will impact my practice.				
Strongly Disagree				Strongly Agree
1	2	3	4	5
4. The activity was presented objectively and free of commercial bias.				
Strongly Disagree				Strongly Agree
1	2	3	4	5
